# A Murine, Bispecific Monoclonal Antibody Simultaneously Recognizing β-Glucan and MP65 Determinants in *Candida* Species

**DOI:** 10.1371/journal.pone.0148714

**Published:** 2016-02-09

**Authors:** Andrea Zito, Carla Bromuro, Giorgia Mandili, Paola Chiani, Alberto L. Horenstein, Fabio Malavasi, Roberto Cauda, Antonio Cassone, Antonella Torosantucci

**Affiliations:** 1 Laboratory of Immunogenetics and CeRMS, Department of Medical Sciences, University of Torino and Transplant Immunology, Città della Salute e della Scienza, Torino, Italy; 2 Istituto Superiore di Sanità, Department of Infectious, Parasitic and Immune-mediated Diseases, Rome, Italy; 3 Department of Molecular Biotechnology, University of Torino, Città della Salute e della Scienza, Torino, Italy; 4 Institute of Infectious Diseases, Catholic University of Rome, Rome, Italy; 5 Center of Functional Genomics, Genetics and Biology, University of Perugia, Perugia, Italy; New York State Dept. Health, UNITED STATES

## Abstract

There is a real medical need of new diagnostic tools for the early recognition of invasive *Candida* infections. We exploited a rather simple and rapid redox methodology to construct a bispecific monoclonal antibody (bsmAb) that combines a monoclonal antibody (mAb) directed against 1,3-β-D-glucan, a well-known, pan-fungal diagnostic biomarker, with a mAb recognizing MP65, a major immunogenic mannoprotein secreted by *C*.*albicans* and other *Candida* species. The bsmAb (MP65/bglu mAb) was successfully produced and purified at high yields and proved to bind and reveal simultaneously, with high sensitivity, the β-glucan and MP65 antigens in both purified and native forms. The MP65/bglu mAb is the first bispecific antibody generated against a fungal microorganism and may prove useful for the concurrent detection of different and clinically significant *Candida* biomarkers in patient sera.

## Introduction

Invasive fungal infections caused by *C*. *albicans* and other *Candida* species are a major cause of morbidity and mortality in hospitalized patients worldwide [1,2]. Despite the recent development and clinical availability of antifungal drugs of high efficacy and reduced toxicity, these infections remain difficult to control and their associated mortality rates are still unacceptably high, approaching 40–50% in some cases. This is further burdened by the high cost of treatment [1,2].

A main obstacle to an efficient clinical management of invasive candidiasis is the lack of diagnostic methodologies allowing the early detection and treatment of the disease [1–5]. Culture-based diagnostics typically are too insensitive and time-consuming. A number of noncultural assays, mostly based on the detection of fungal biomarkers in patient circulation, are making their way into the medical setting or are currently under investigation, but none have yet proved sufficiently sensitive and specific to detect the disease at a time when fungal burden is still relatively low and more responsive to antifungal therapy.

Several studies on the impact of treatment delays due to the time required for diagnosis show that patient mortality and hospitalization costs increase significantly for each day without anti-fungal therapy [3–5]. Clinicians and microbiologists agree that reliable tools for the early recognition of the fungal infection are a strong medical need [2], either as novel strategies for enhancing the sensitivity and specificity of current diagnostic methods or as new diagnostic assays.

Monoclonal antibodies (mAbs) are the gold standard for early detection of diagnostically relevant antigens. Highly specific and easily standardized, mAbs can reveal just picograms per milliliter of the specific target. The possibility of combining two pre-existing mAbs into one bispecific antibody (bsmAb)—a dual-Fab molecule recognizing both ligands of the mAbs of origin—has long been known [6–10]. To date, bsmAbs have been mostly investigated as candidate dual targeting therapeutics for treatment of cancer or inflammatory diseases; the potential of these reagents in the diagnosis of infectious diseases has remained almost unexplored [11,12].

The working hypothesis of this study is that the development of bsmAbs for the simultaneous detection of two different and significant *Candida* biomarkers would make it possible to design highly specific and sensitive assays for use in diagnosing invasive *Candida* infections. Our investigation exploited bsmAb technology to combine an anti-laminarin mAb, recognizing 1,3-β-D- and 1,6-β-D-glucan sequences, with a mAb directed against MP65, an immunodominant mannoprotein of *C*. *albicans* [13–16].

## Materials and Methods

### MAb Production and Purification

MAb 2G8, an anti-laminarin mouse IgG_2b_ [13,14], and 4C8, a murine IgG_1_ recognizing the protein moiety of the MP65 mannoprotein of *C*. *albicans* [15,16] were produced from culture supernatants of respective hybridoma cell lines. These were concentrated using a QuixStand benchtop system (GE Healthcare, Milano, Italy) equipped with a 30-kDa cutoff membrane and purified by high performance liquid chromatography (HPLC) on a rProtA MabSelect Sure^™^ column (GE Healthcare). To this aim, the concentrated supernatants were added with 112.6 g/l glycine, 175 g/l NaCl, and 3 g/l NaOH to achieve a pH of 8.9 and loaded onto the column at a 2 ml min^−1^ flow rate. Bound Igs were eluted with 3 column volumes of 0.1 M sodium citrate buffer, pH 5.8 (mAb 4C8) or pH 3.5 (mAb 2G8) and concentrated/diafiltrated against 10 mM sodium phosphate buffer, pH 6.7 using a Centricon Plus-80, 30-kDa MWCO, concentrator (Millipore, Milano, Italy) [17]. The samples were then processed by preparative chromatography on a Hydroxyapatite (HA) column (Bio-Rad, Milano, Italy) and eluted with a 110 min-linear gradient from 10 to 400 mM sodium phosphate buffer, pH 6.7, at a flow rate of 2 ml min^−1^. The pooled HA peak fractions containing the mAbs were concentrated and extensively dialyzed against phosphate-buffered saline (PBS), pH 7.2, using a C80 Centricon Plus (Millipore), and finally filtered through a 0.22-μm disposable hydrophilic Posidyne membrane syringe filter (Pall, Ann Arbor, MI, 25-mm diameter).

The IgG concentration was monitored and measured by absorbance at 280 nm throughout the entire process. Evaluation of retention times, peak heights and relative peak areas were made using the Gold Beckman software [18].

### Redox Methodology

The 2G8/4C8 bsmAb was prepared by adapting the redox method described by Carling et al. [19]. To reduce the parental mAbs to monovalent antibody (mvAb), 2-mercaptoethanesulfonic acid sodium salt (Sigma-Aldrich, USA) was diluted in H_2_O and added to 1 mg of mAb 2G8 or to 1 mg of mAb 4C8, both diluted in PBS, pH 7.2, at the final concentration of 10 mM (mAb 2G8) or 30 mM (mAb 4C8). The samples were incubated at 37°C for 25 min. Re-association of mvAbs into bsmAbs was induced by mixing the reduced 2G8 and 4C8 mAbs (1:1 ratio), followed by oxidation through dialysis against 3 buffer exchanges of PBS, pH 7.2 (24 h at 4°C).

### Purification and Characterization of the bsmAb

The bsmAb was purified by a two-step chromatography separation onto an HA column, after careful selection of appropriate fractionation gradient. Purification was performed at a flow-rate of 2 ml/min using 10 mM phosphate buffer, pH 6.7 as buffer A and 400 mM phosphate buffer, pH 6.7 as buffer B [18]. The eluted fractions from the first HA column were dialyzed against PBS and subsequently re-purified in a second HA column to isolate the peak containing the 2G8/4C8 bsmAb. The peak fractions corresponding to the active bsmAb were pooled, dialysed against sterile PBS and stored at -20°C.

SDS-PAGE analysis of purified bsmAb and parental mAbs was performed onto 10% polyacrylamide gels under reducing and non-reducing conditions, according to standard techniques [20]. Isotype class and subclass were analyzed by a standard immunodiffusion test [21], using goat anti-mouse IgG_1_, IgG_2a_, IgG_2b_, IgG_3_ and IgM antibodies, respectively (SouthernBiotech, Birmingham, AL).

2D-IEF analysis was performed using ready-made immobilized pH gradient (IPG) strips (7-cm IPG strips, pH 3–10). Each sample (250 μg of protein for preparative gels) was applied onto an IPG strip by in-gel rehydration for 20 h, with the addition of dithiothreitol (DTT) and Ampholine pH 3–10. IEF was carried out in a Protean IEF cell apparatus (Bio-Rad, Segrate, Italy). Focusing was performed at 18°C with a limit of 50 mA per strip. Subsequently, IPG strips were equilibrated for 15 min in 6 M urea, 2% SDS, 0.05 M Tris-HCl pH 8.8, 20% glycerol, 1% DTT buffer and for 12 min in 6 M urea, 2% SDS, 0.05 M Tris-HCl pH 8.8, 20% glycerol, 2.5% iodoacetamide buffer. For the second dimension, strips were run into 10% acrylamide gels in a Mini Protean system (Bio-Rad). Gels were stained with colloidal Coomassie blue G-250. Image analysis was performed using the PD-Quest software (version 7.2, Bio-Rad), according to the manufacturer’s instructions. Normalization of each individual spot was performed according to the total quantity of the valid spots in each gel, after subtraction of the background values. The spot volume was used as the analysis parameter to quantify protein expression.

### Antigens

Laminarin was purchased from Sigma-Aldrich, USA. Recombinant MP65p, expressed in *E*.*coli* on the basis of the published sequence [22] and purified from bacterial culture supernatants, was produced by GeneScript, Piscataway, NJ. Concentrated and dyalized culture supernatants containing the antigenic material secreted by plastic-adherent, hyphal form-growing *C*.*albicans* (SAM) were obtained as described previously [13].

Biotin labelling of laminarin was performed essentially as described by Wang et al. [23]. Beta-1,6-linked, branch residues of the polysaccharide were oxidized by sodium metaperiodate (Sigma-Aldrich) and newly formed aldheyde groups were reacted with ethylenediamine (Sigma-Aldrich). Resulting Schiff base was stabilized by reduction with sodium borohydride (Sigma-Aldrich) and the aminated polysaccharide was finally reacted with the *N*-hydroxysuccinimide ester of biotin (BNHS, Sigma-Aldrich) and purified by precipitation with 95%, v/v ethanol in the cold and repeated washings. MP65p was biotinylated by direct reaction with BNHS in 1M carbonate buffer, pH 8.3 (ratio protein to BNHS, 10:1) and purified by diafiltration against PBS buffer in a 10 kDal MWCO ultrafiltration device (Millipore, Milano, Italy).

### Analytical Determinations

Polysaccharide or protein content in the preparations of purified mAbs, bsmAb, biotinylated antigens or SAM were determined by the phenol-sulphuric acid method or by the MicroBCA Protein assay (Bio-Rad Laboratories), respectively, as previously reported [14].

### Immunoenzymatic Assays

In indirect ELISA experiments, the various antigens were diluted in 0.05 M carbonate/bicarbonate buffer, pH 9.6 (laminarin, 50 μg/ml; MP65p, 5.0 μg/ml; SAM, 5.0 μg/ml protein, unless otherwise specified) and adsorbed onto polystyrene microtiter plates (MaxiSorp; NUNC). Plates were blocked with 3% bovine serum albumin (BSA, Fraction V, Sigma-Aldrich) in PBS and reacted with two-fold dilutions of the parental mAbs or bsmAb, followed by alkaline phosphatase-conjugated, anti-mouse IgG antibody (Sigma-Aldrich) and the enzyme substrate (p-nitrophenyl phosphate disodium, Sigma-Aldrich), as previously described [13]. Absorbance was read at 405 nm and readings from negative controls (wells non reacted with the mAbs or reacted with an irrelevant mAb) were subtracted from all absorbance values.

In capture ELISA, MaxiSorp plates were coated with an Fc-specific, goat anti-mouse IgG antibody (5 μg/ml in PBS, on, +4°C), washed and reacted with the bsmAb at the desired dilutions in PBS (1h, 37°C). After thorough washings and blocking with 3% BSA in PBS, the plate-bound bsmAb was reacted with different concentrations of biotinylated laminarin or MP65p for 1h at 37°C. Wells were washed again and the amount of bound antigen was evaluated by the addition of horse-radish peroxidase-conjugated streptavidin (Sigma-Aldrich) followed by 3,3′,5,5′-tetramethylbenzidine-H_2_O_2_ substrate solution (1-step Ultra-TMB ELISA, Thermo Scientific, USA). Wells were acidified by addition of 1M H_2_SO_4_ and absorbance read at 450 nm. Negative control wells (non reacted with the bsmAb or with the biotinylated antigens) were always run in parallel to evaluate background O.D. values.

### Statistics

Linear regression analysis of bsmAb binding curves and Student’s t test were performed using the Prism 4 software (GraphPad, USA).

## Results

### Parental mAbs

To develop the MP65/bglu mAb we selected two mAbs that specifically recognize two different, disease-relevant, fungal antigens. The first (mAb 2G8) is a murine IgG_2b_ specific for β-1,3-glucan, a cell wall component expressed and secreted by nearly all fungal pathogens [13,14]. The second mAb (4C8) is a mouse IgG_1_ that recognizes the protein moiety of MP65 (MP65p), a highly immunogenic mannoprotein involved in *Candida* cell wall maintenance and remodeling [15,16,24].

The parental mAbs were massively produced in cell cultures, and supernatants underwent all the steps for purification as described [17]. The purified mAbs maintained the purity grade and binding efficiency shown by reference mAb preparations, as assessed by SDS-PAGE analyses and ELISA testing (data not shown).

### Production of the bsmAb

**Fig 1:**
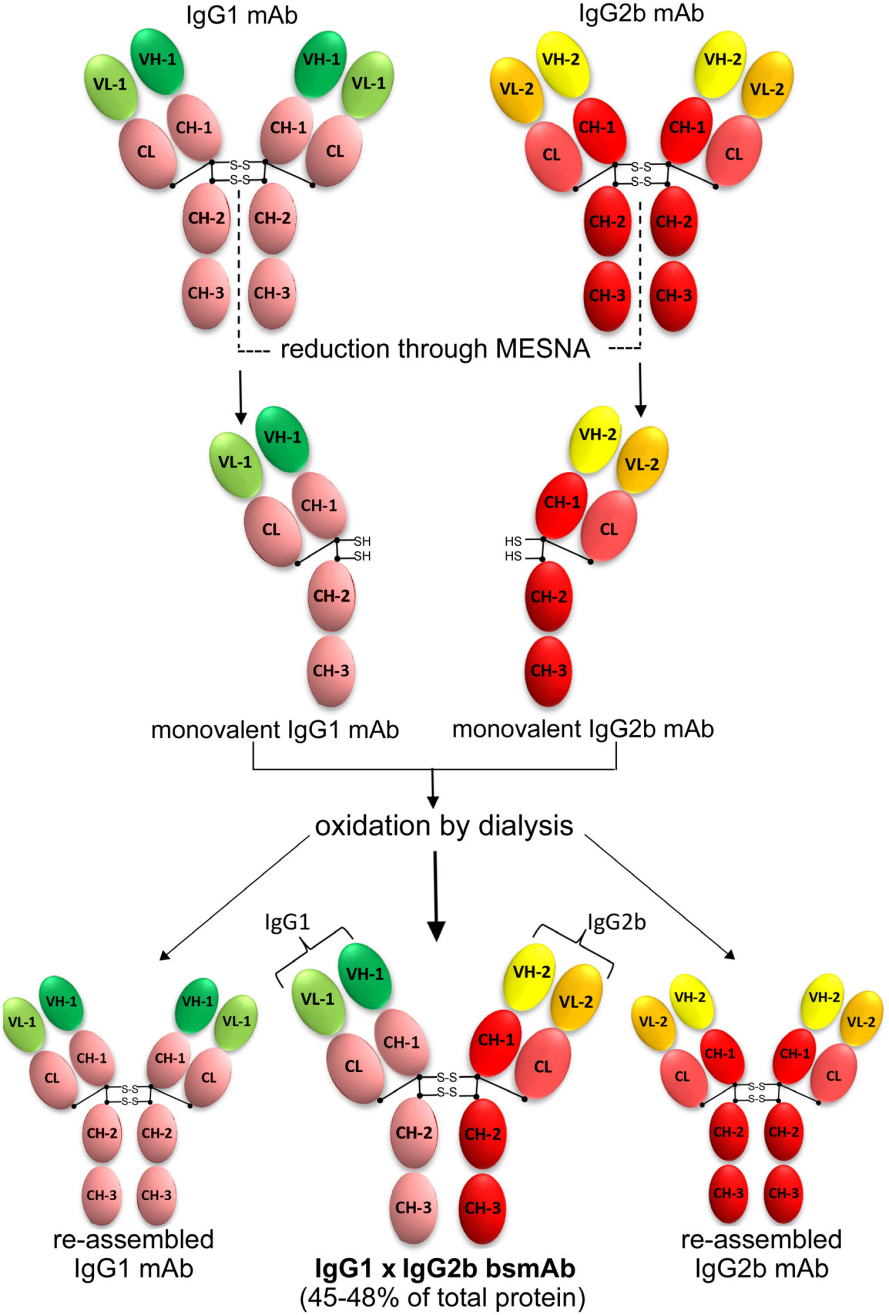
Schematic diagram of MP65/bglu mAb generation by the redox method. Modified from: Carlring et al., PLoS One. 2011;6: e22533.

The construction of the MP65/bglu mAb relied upon a rapid method based on reduction-oxidation of the parental IgGs [19]. As illustrated in Fig 1, the method exploits the mild reducing agent 2-mercaptoethanesulfonic acid (MESNA) to break the inter-heavy chain bonds of the parental IgGs and to reduce them to monovalent mAbs (mvAbs). Subsequent dialysis under oxidizing conditions provided re-associations of the mvAbs to form bsmAb molecules.

**Fig 2:**
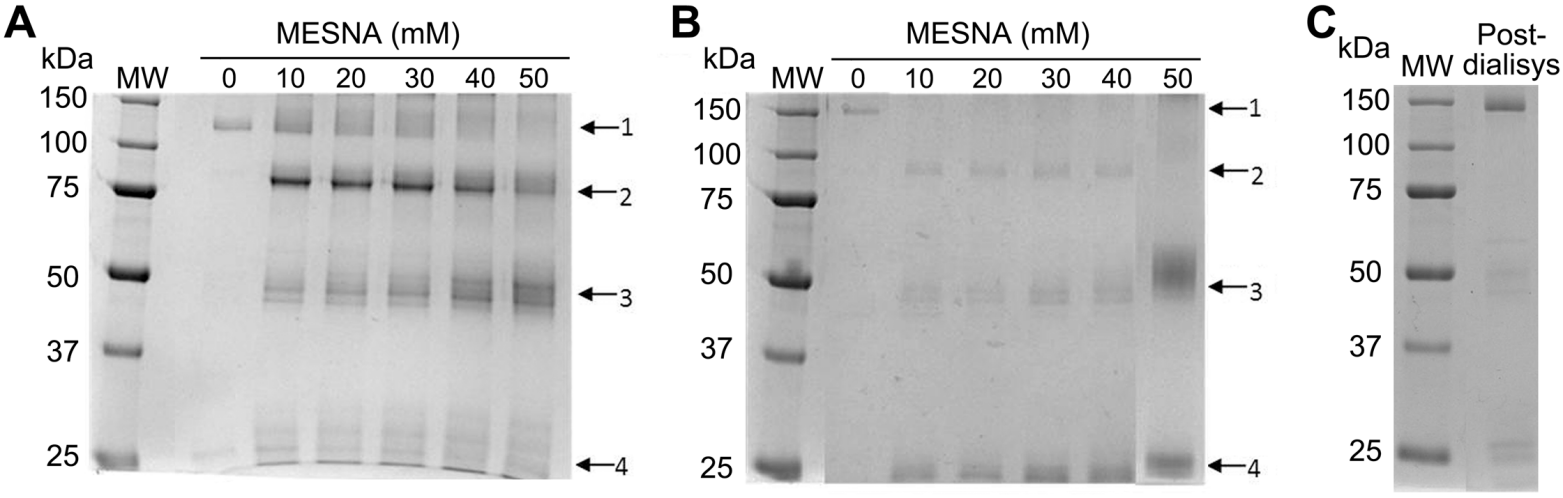
Tuning redox methodology for MP65/bglu mAb construction. Panels A and B show the efficiency of different MESNA concentrations (0 to 50 mM, as indicated) in splitting mAb 2G8 (A) or mAb 4C8 (B) into mvAb fragments, as evaluated by their SDS-PAGE profiles under non-reducing conditions. The arrows indicate the presence of the whole mAb (1), mvAb (2), heavy chain (3), and light chain (4). Panel C shows a non-reducing SDS-PAGE analysis of the 2G8/4C8 mvAb mixture after oxidation by dialysis, demonstrating the re-association of mvAb fragments into whole Ig molecules. MW = molecular weight standards.

Preliminary experiments were performed to establish the optimal conditions of MESNA treatment for obtaining mvAbs from the parental 2G8 and 4C8 IgG. Different concentrations of MESNA and different incubation times were used to reduce the purified mAbs. The composition of the resulting products was then analyzed by non-reducing SDS-PAGE. The optimal reduction of the 2G8 mAb was obtained at a MESNA concentrations of 10 mM, as indicated by the presence of a sharp mvAb band at ~75 kDa in the electrophoretic profile of the final reaction mixture (Fig 2A). Instead, 30 mM MESNA yielded the highest amount of mvAb fragments, with a molecular weight of ~85 kDa, from the 4C8 mAb (Fig 2B). Both reduced mAb preparations showed only a faint band attributable to the intact mAb, confirming their high susceptibility to reduction at the conditions adopted (Fig 2A and 2B). MESNA reduction also led to the release of small amounts of free heavy and light mAb chains, which were almost totally removed by subsequent dialysis (Fig 2). These experiments defined the optimal incubation conditions for MESNA treatment were exposure of IgG at 37°C for 25 min (data not shown).

Reassembling of mvAbs into bsmAbs was initiated by mixing equal amounts of reduced 2G8 and 4C8 mvAbs, followed by oxidation through dialysis against PBS [19]. SDS-PAGE analysis of the mvAbs mixture after the dialysis step showed an almost complete re-assemblage of the immunoglobulins, as shown by the presence of a major band at 150 kDa (Fig 2C). MvAbs re-associate randomly during the oxidizing reaction, hence the 150 kDa product represented a mixture of re-assembled original mAbs and newly formed bsmAb molecules (Fig 1).

### MP65/bglu mAb Purification

**Fig 3:**
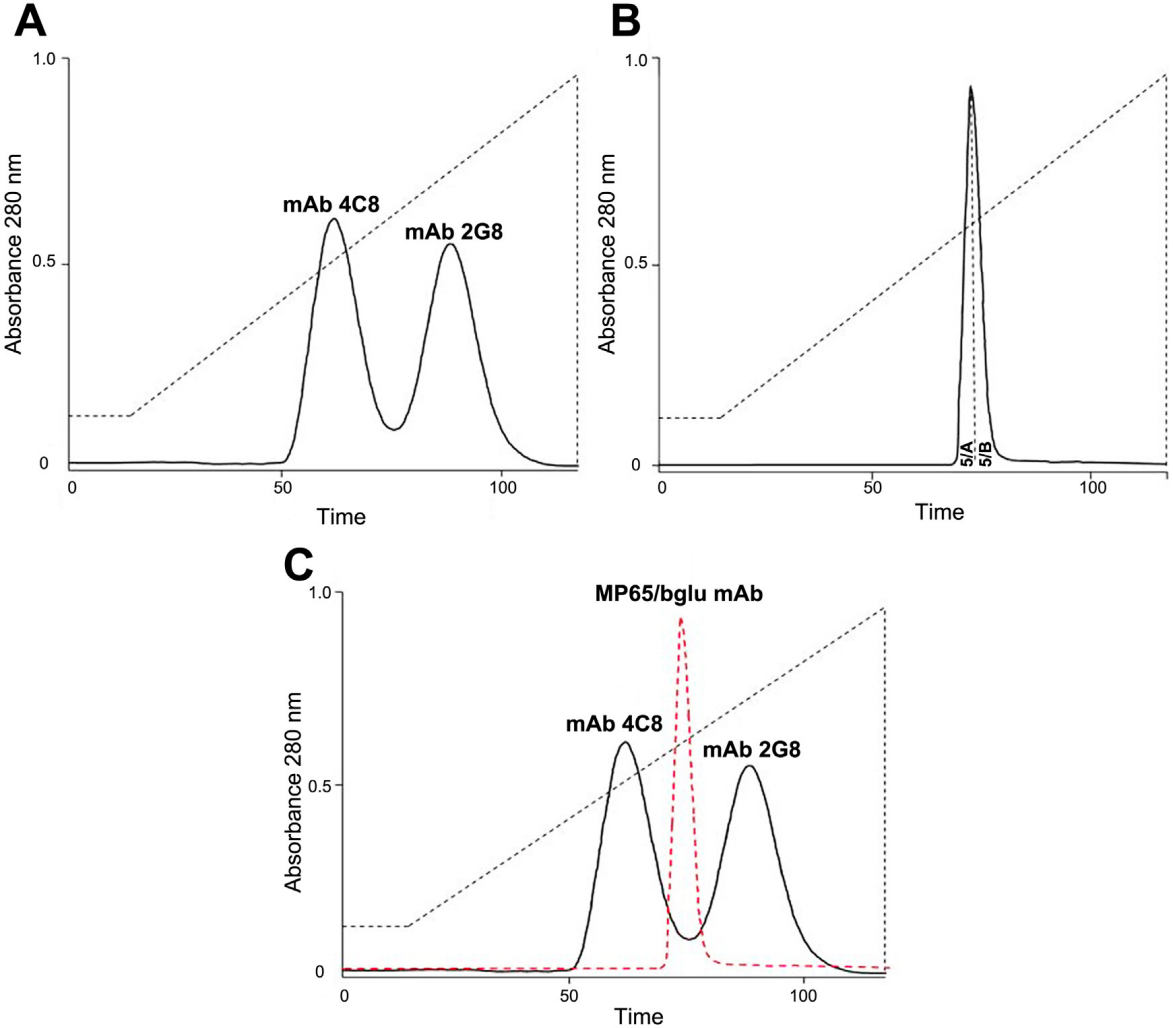
Purification of the MP65/bglu mAb by HA-ion-exchange chromatography. Elution profiles of the 2G8 and 4C8 mAbs (Panel A), the 2G8 x 4C8 bsmAb (Panel B) and merge of all three profiles (Panel C); in C, the bsmAb peak is indicated by the dotted, red line. Chromatographic separation was performed on a 50 x 25 mm I.D. HA column at a flow rate of 2 ml/min. A 110 min linear gradient from 10 to 400 mM phosphate (dotted black line) was used. In B, dotted line inside of the bsmAb elution peak shows its separation into the two fractions 5/A and 5/B.

The bsmAb was composed by two different and monovalent IgG moieties (mvIgG), each of which corresponded to one half of the parental IgGs (Fig 1). The resulting total net charge of this molecule (mvIgG_1_ x mvIgG_2b_) was thus different from that of the parental mAbs (mvIgG_1_ x mvIgG_1_ or mvIgG_2b_ x mvIgG_2b_). HPLC on hydroxyapatite (HA) led to an efficient separation of the MP65/bglu mAb and the re-assembled 2G8 or 4C8 mAbs according to their individual charges [25]. Successful purification of the bsmAb, however, critically required the choice of gradients of appropriate ionic strength for the chromatographic separation, as to obtain significant differences in the retention times of the bsmAb and the parental mAbs. Gradients were selected by careful evaluation of the retention times of the parental mAbs in different conditions. As shown in Fig 3, HA chromatography allowed the purification of a definite peak attributable to the bsmAb, distinct from those originated by the parental 2G8 and 4C8 mAbs.

To verify the identity of the MP65/bglu mAb peak, fractions corresponding to the ascending and descending part were collected separately and referred to as 5/A (ascending peak) and 5/B (descending peak) fraction (Fig 3C).

**Fig 4:**
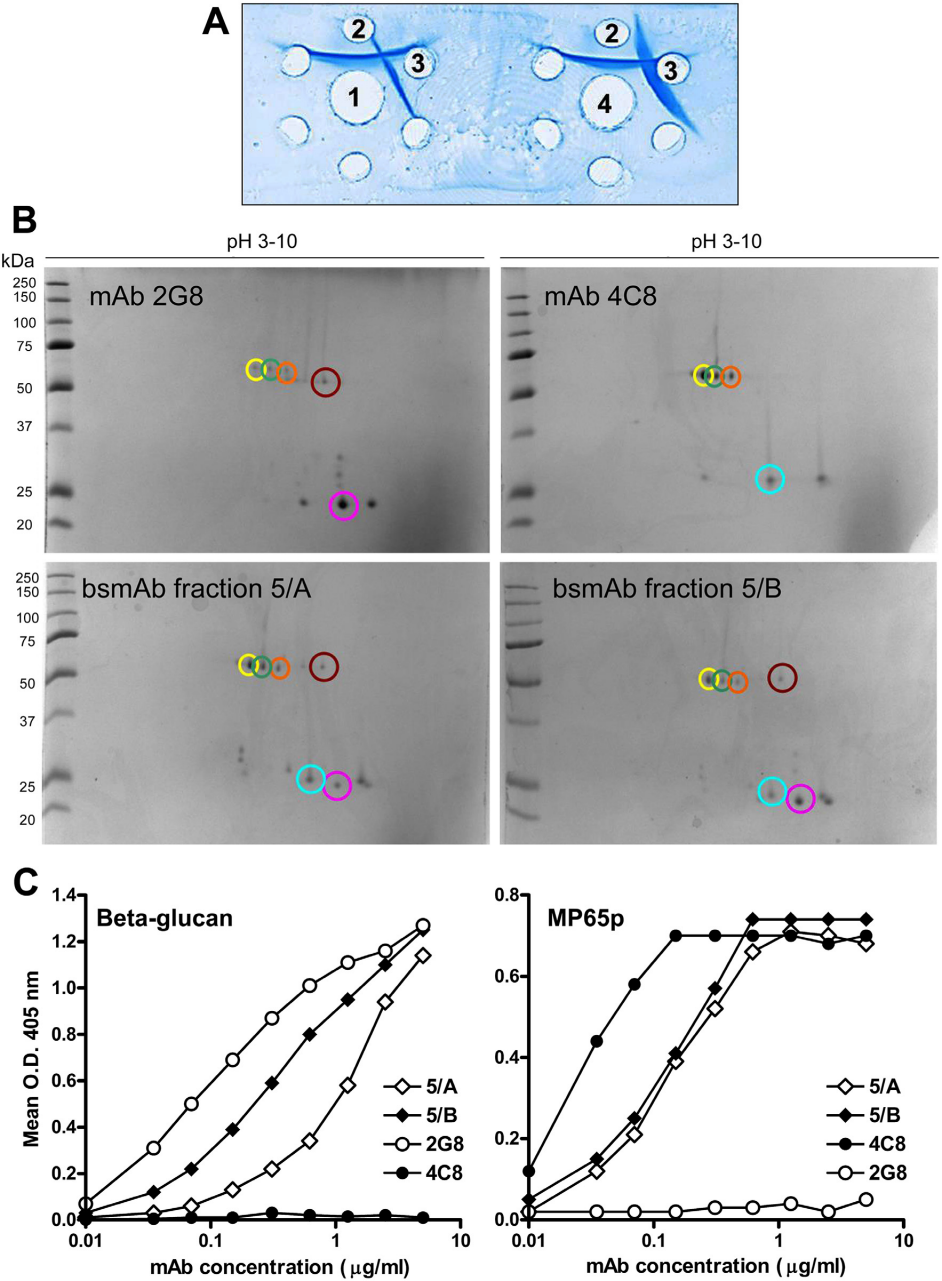
Characterization of the 5/A and 5/B bsmAb fractions. **Panel A. Reactivity of 5/A and 5/B in the Ouchterlony double immunodiffusion assay.** Central well 1 was loaded with fraction 5/A and central well 4 with fraction 5/B. Side well 2 contained a goat, anti-mouse IgG_1_ mAb and side well 3 a goat anti-mouse IgG_2b_ mAb. **Panel B. 2D-IEF analysis of the bsmAb fractions in comparison with the 2G8 and 4C8 parental mAbs.** IEF separation was performed as described in Materials and Methods. Image analysis of the stained gels was performed using the PD-Quest software. Circle of the same color indicate corresponding protein spots in different mAb samples. **Panel C. Binding of the bsmAb fractions to β-glucan and MP65p.** Laminarin or MP65p adsorbed onto ELISA plates were reacted with fraction 5/A or 5/B or with the parental mAb 2G8 or 4C8, at the concentrations indicated. Binding of the Abs was revealed by an alkaline phosphatase-conjugated, anti-mouse IgG mAb followed by p-nitrophenyl phosphate disodium as the enzyme substrate. Binding is expressed as the mean, O.D. 405 nm readings from triplicate wells after subtraction of O.D. from the negative controls (wells with irrelevant Abs). The SD of all triplicate determinations was always <20% and is not shown in the graph.

Classical Ouchterlony tests (Fig 4A) clearly showed that the two fractions were recognized by both anti-IgG_1_ and anti-IgG_2b_ antibodies. Precipitin lines were of similar intensity, confirming that 5/A and 5/B were both a combined IgG_1_ x IgG_2b_ complex.

2D-IEF analysis showed that the fingerprints of the two fractions were almost identical in quantity and quality and substantially corresponded to the combination of the individual fingerprints exhibited by the parental mAbs (Fig 4B).

Finally, the fractions were assayed for reactivity with laminarin, a standard β-1,3 glucan polysaccharide, or with recombinant MP65p, used as solid-phase antigens in ELISA. The parental 2G8 and 4C8 mAbs were tested at the same concentrations as reactivity reference standards. The results demonstrated that both 5/A and 5/B were able to recognize plastic-adsorbed laminarin or MP65p, in a dose-dependent fashion (Fig 4C). Comparison of the binding efficiency of the two fractions, as exemplified by the final assay outputs produced (O.D. 405 nm), indicated an almost identical reaction patterns with MP65p and a slightly more efficiency in binding laminarin by 5/B (Fig 3C). However, the reactivity of the bsmAb fractions was comparable to that exhibited by the parental mAbs, at least at the highest doses.

Based on these results, subsequent experiments were performed using the entire bsmAb peak.

### Validation of the bsmAb

Definitive validity of the construct was investigated by testing its ability to bind simultaneously and independently the epitopes recognized by the parental mAbs and by evaluating whether, and at what level of sensitivity, the MP65/bglu mAb was able to capture the free target antigens in solution. This was critical to envision the potential of the bsmAb as a single, specific reagent able to reveal as a whole both the β-glucan and MP65p diagnostic antigens in the sera of *Candida* infected patients.

**Fig 5:**
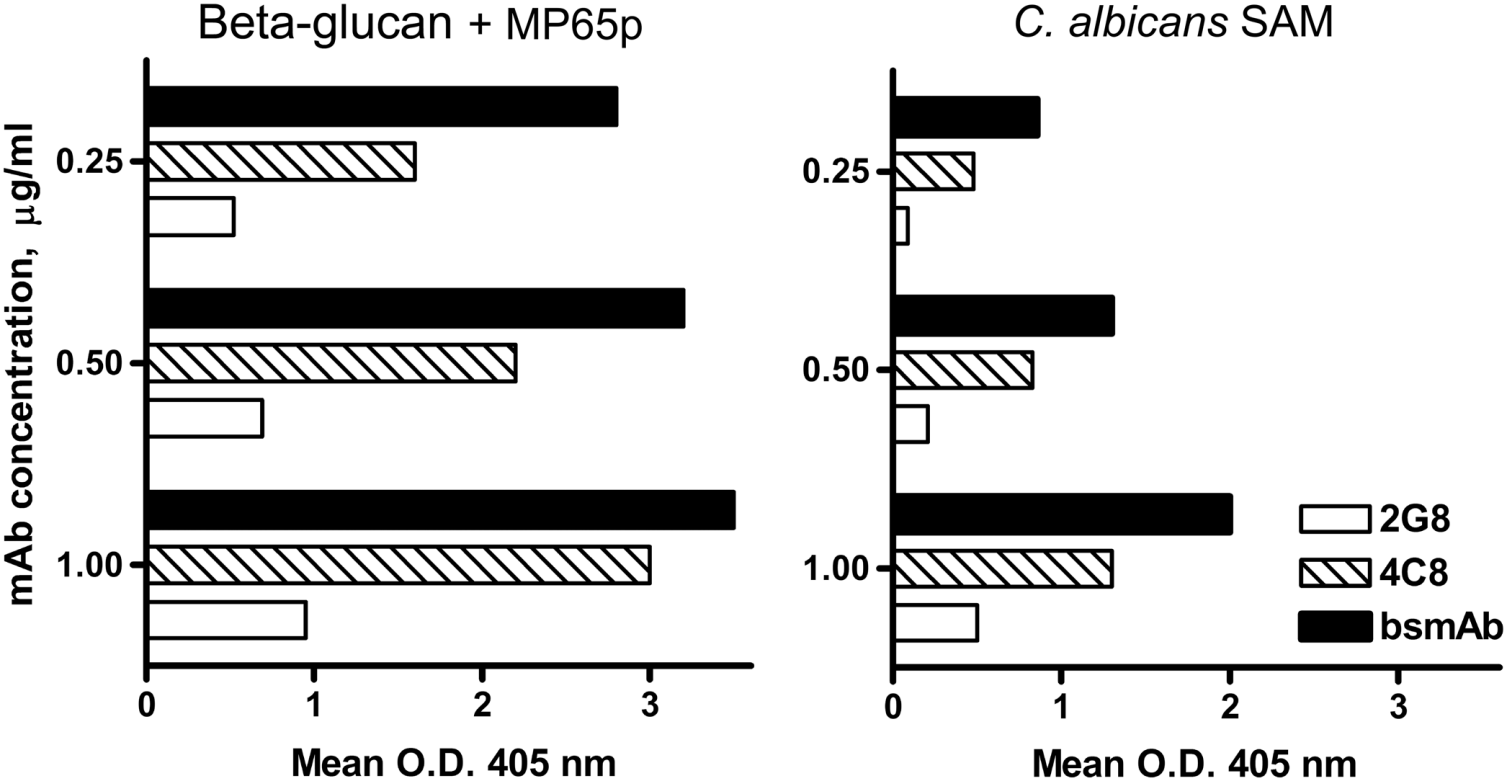
The bsmAb is able to bind contemporaneously and independently the β-glucan and MP65p antigens. The ELISA plates were coated with a mixture of laminarin and MP65p (20 and 5.0 μg/ml, respectively) or with a concentrated culture supernatant of *C*.*albicans* hyphae (SAM, 5.0 μg/ml protein) containing naturally secreted β-glucan and MP65. The graph shows a comparison of mean O.D.405 nm values resulting upon reaction of the antigen mixtures with mAb 2G8, mAb 4C8 or bsmAb 2G8X4C8, at the indicated concentrations. SD between replicates was always <20% and is not reported.

First, the MP65/bglu mAb was assayed in indirect ELISA experiments against a combination of the antigens recognized by the parental mAbs as the plastic-adsorbed target (Fig 5). The combination was either a mixture of laminarin and recombinant MP65p or a concentrated culture supernatant of hyphal form-growing *C*.*albicans* (SAM), a preparation that is known to contain both the β-glucan and MP65 antigens as they are naturally expressed and secreted by the fungus [13,16,26]. Results from these experiments showed that reaction of the bsmAb with both the laminarin-MP65p mixture (Fig 5A) or the native fungal SAM (Fig 5B), at all doses tested, produced final O.D. readings approximately equivalent, if not slightly higher, to the sum of those produced by each parental mAb against its respective antigen. This clearly indicated that bsmAb could indeed bind the β-glucan and Mp65 antigens simultaneously and independently, as expected, with no loss of functional affinity with respect to the mAbs of origin and even in the form they are natively produced by fungal cells.

In all conditions tested, O.D. readings generated by the bsmAb were significantly higher (P<0.01, Student’s t test) than those generated by the mAb 2G8. Differences between readouts produced by the bsmAb and the mAb 4C8 were always statistically significant (P<0.05, Student t test) except when the two mAbs were compared at the concentration of 1μg/ml against the laminarin-MP65p mixture.

**Fig 6:**
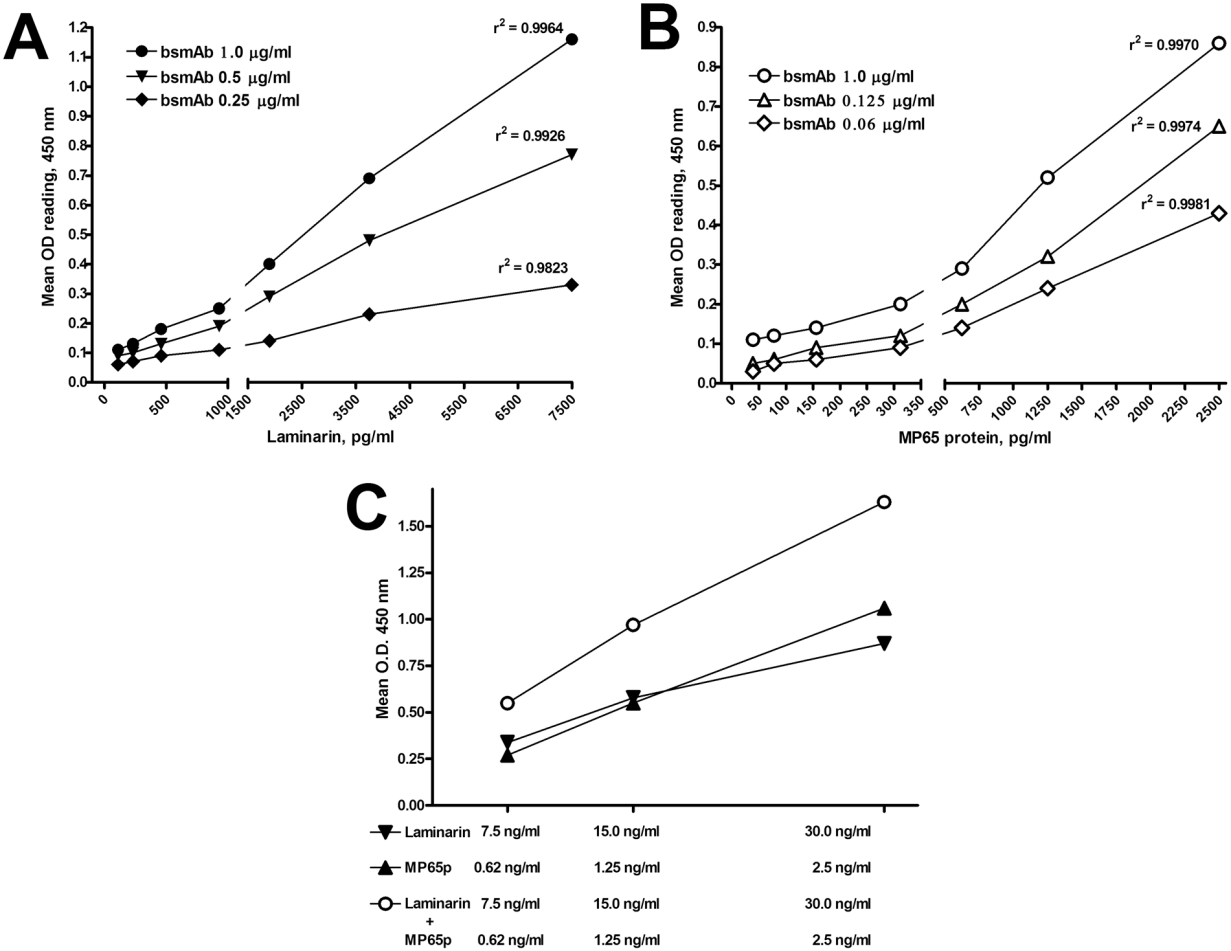
Antigen capture by the MP65/bglu mAb. **Panel A and B. Dose-response, β-glucan and MP65p capture curves.** An Fc-specific, goat anti mouse IgG coating was used for the oriented binding of the bsmAb, at various concentrations, to the ELISA plates. Plate-bound bsmAb was then reacted with different doses of biotinylated laminarin (Panel A) or MP65p (Panel B). The amount of captured antigens was revealed by the addition of peroxidase-conjugated streptavidin followed by the enzyme substrate. O.D. 450 nm readings are the mean from duplicate wells after subtraction of O.D. values from the negative controls (wells with no antigen or no bsmAbs). R^2^ values of the resulting capture curves were calculated by linear regression analysis. **Panel C. Dual antigen capture by the bsmAb.** The ELISA plate-bound bsmAb (0.2 μgml) was reacted in comparison with different doses of biotinylated laminarin or biotinylated MP65p or with a mixture containing both biotynilated laminarin and MP65p. The ELISA assay was performed as described above for data in Panels A and B.

Following these experiments, we constructed ELISA assays in which the bsmAb was bound to the plate and evaluated for its ability to capture and reveal biotin-labelled laminarin or MP65p in solution. Checkerboard titration experiments showed that the bsmAb was efficient at capturing both antigens (Fig 6A and 6B). The outputs (final O.D. readings) of the assays were MP65/bglu.mAb- and antigen-dose dependent. Further, a good linearity was found for antigen dose-O.D. response curves obtained at all the different bsmAb concentrations assayed, with r^2^ values always approximating 1.0 (Fig 6A and 6B). Finally, the output of the experiments was readable at antigen concentrations as low as 50 pg/mL^-1^ (laminarin) or 150 pg/mL^-1^ (MP65p), demonstrating that the MP65/bglu mAb had a high avidity for both fungal biomarkers (Fig 6A and 6B). In other experiments (Fig 6C), the ELISA plate-bound MP65/bglu mAb was also reacted in parallel with different doses of biotinylated laminarin or MP65p, separate or mixed toghether. Similarly to what observed previously (see Fig 5), O.D. readings resulting upon capture of the antigen mixture roughly approximated the summed values generated by the capture of each single antigen, indicating that β-glucan and MP65p did not exert any mutual steric hindrance or generic interference in binding to the bsmAb (Fig 6C).

Overall, these results suggested that the MP65/bglu mAb was very efficient and sensitive for the synchronous capture and revelation of the two fungal biomarkers.

## Discussion

Despite the huge potentialities of bsmAbs for the detection and treatment of cancer and other human diseases, only few examples of bsmAb devised for anti-bacterial or anti-fungal therapy are reported in current literature [27–32]. BsmAbs proposed for the diagnosis of human microbial infections are even fewer, and were all constructed to bind in combination a microbial antigen and various types of reporter molecule, in order to improve antigen recognition specificity in different immunoassays [33–35].

We describe here the development and characterization of a novel, anti-*Candida* bsmAb (MP65/bglu mAb) designed for the dual-detection of two diverse, diagnostically relevant, fungal biomarkers in patient sera. The bsmAb was constructed from two previously described murine mAbs [13,14,16], that recognize two *Candida* antigens whose levels are known to increase in patient circulation in the course of the fungal infection. One of them is the pan-fungal polysaccharide β-glucan, a well-established disease biomarker that is currently assayed for the diagnosis of infections caused by different fungi, including *Candida* [36]. The companion antigen, MP65p, is a protein originally identified by us in *C*.*albicans* as the main fungal antigen targeted by the human cellular immune responses [15,16,37]. MP65p is secreted at very high levels, *in vivo* and *in vitro*, particularly by *Candida* hyphae which are the form of growth generally associated to infectious candidiasis [24,26]. Close homologues of this protein are also expressed and secreted by most of the pathogenic *Candida* species, such as *C*. *guilliermondii*, *C*. *glabrata*, *C*. *parapsilosis* and *C*. *tropicalis* [22]. Building upon our previous work, other authors have more recently proposed MP65p as a valid marker of *Candida* infection, although it is not yet used in clinical diagnostics [24].

Traditional methods of producing bsmAb rely on immunoglobulin G (IgG) heterocross-linking or the production of hybrid hybridomas [25]. Alternative methods, such as Fc-engineering [38], chemical redox [19,39] or both [40–42], have been under development in the last few years. Hybrid hybridomas and engineering are highly sensitive as well as consistent, but they are time consuming and expensive. To construct the MP65/bglu mAb, we applied a manageable chemical redox methodology that has proven successful and time-saving for obtaining different types of bsmAbs, and that is particularly suitable for the production of bsmAb reagents for use in early pilot studies. Our results show that by coupling the redox strategy with HA purification, the production of bsmAbs from homospecific immunoglobulins of different isotypes is both simple and efficient. We were able to produce and purify the bsmAb with yields of up to 50%, which is twice that previously reported [19].

The bsmAb generated could simultaneously bind with high affinity the β-glucan and MP65p antigens. Indeed, both ELISA experiments with plastic-adsorbed antigen mixtures and ELISA dual-capture assays showed that the MP65/bglu mAb produced readout values that were essentially the sum of those individually produced by the two mAbs of origin. These results also indicate that, at least in our assay conditions, the overall avidity of the two bsmAb binding arms is preserved, if not increased, with respect to the two entire parental mAbs suggesting that assemblage of the 2G8 and 4C8 mAbs could enhance the affinity and/or avidity of one or both antigen binding sites in the resulting bispecific Ig. This might be caused by the structural diversity of the constant regions of the MP65/bglu mAb, that is a combined IgG1xIgG2 molecule, unlike the IgG1 and IgG2 mAbs of origin. In fact, it is well known that variations of the constant regions can significantly impact the affinity and even the fine specificity of Abs with identical antigen binding sites [43,44]. Moreover, an altered avidity of bsmAbs as compared to parental mAbs has been reported by several authors [45–47].

ELISA capture curves revealed a high efficiency of the MP65/bglu mAb in detecting the fungal antigens. In particular, the sensitivity of the bsmAb effectively detected either β-glucan or MP65p at very low concentrations, such as those that are present and considered diagnostically significant in *Candida*-infected patients [24,36].

The standard β-glucan (laminarin) and the recombinant MP65p used in this investigation are mock antigens that express the epitopes recognized by the bmAb parental Igs but are structurally different from the native fungal antigens. Fungal β-glucan, for instance, is much more extensively branched than laminarin, while MP65p is released by fungal cells in a heavily mannosylated form. Therefore, the ability to recognize naturally released β-glucan and MP65p in hyphal secretion, a material that mimics the antigenic secretion produced by the infecting fungus *in vivo*, is another important and diagnostically relevant feature of our MP65/bglu mAb.

Early serological diagnosis of invasive candidiasis remains a real challenge. Specific approaches already attempted in this field have included the detection of fungal DNA using PCR-based methods or the analysis of circulating fungal biomarkers in patient sera [36]. PCR methodologies mostly proved difficult to standardize and are as yet experimental. Instead, commercial assays of 1,3-β-D-glucan or α-1,2-D-/α-1,6-D-mannan, two cell wall polysaccharides which increase in serum levels in the course of infection, are now commonly used in clinics [36]. These, however, have multiple drawbacks in terms of sensitivity and specificity and sometimes must be ran in combination to consolidate their diagnostic significance [36].

We consider that the MP65/bglu mAb produced in this work has the potential to contribute to the development of new, “two-in-one” biomarker assays for an earlier and more reliable diagnosis of candidiasis and to open the way to the use of bsmAbs as novel and potent reagents for the sensitive detection of infection biomarkers.
